# Time-resolved plasmon-assisted generation of optical-vortex pulses

**DOI:** 10.1038/s41598-023-41606-3

**Published:** 2023-09-07

**Authors:** Esra Ilke Albar, Franco P. Bonafé, Valeriia P. Kosheleva, Sebastian T. Ohlmann, Heiko Appel, Angel Rubio

**Affiliations:** 1 Max Planck Institute for the Structure and Dynamics of Matter, Luruper Chaussee 149, 22761 Hamburg, Germany; 2 Max Planck Computing and Data Facility, Gießenbachstr. 2, 85748 Garching, Germany; 3 Center for Computational Quantum Physics (CCQ), The Flatiron Institute, 162 Fifth Avenue, New York, NY 10010 USA; 4 Nano-Bio Spectroscopy Group, Departamento de Física de Materiales, Universidad del País Vasco, 20018 San Sebastian, Spain

**Keywords:** Nanophotonics and plasmonics, Ultrafast photonics

## Abstract

The microscopic mechanism of the light-matter interactions that induce orbital angular momentum (OAM) in electromagnetic fields is not thoroughly understood. In this work, we employ Archimedean spiral vortex generators in time-resolved numerical simulations using the Octopus code to observe the behind-the-scenes of OAM generation. We send a perfect circularly-polarized plane-wave light onto plasmonic optical vortex generators and observe the resulting twisted light formation with complete spatio-temporal information. In agreement with previous works, we find that emission from the plasmonic spiral branches shapes the vortex-like structure and governs the OAM generation in the outgoing electromagnetic field. To characterize the generated beam further, we emulate the emission from vortex generators with current emitters preserving the spiral geometry. We subject a point-particle system to the generated field and record the orbital angular momentum transfer between the electromagnetic field and the point particle. Finally, we probe the OAM density locally by studying the induced classical trajectory of point particles, which provides further insight into the spatio-temporal features of the induced OAM.

## Introduction

The investigation of the interaction between twisted- or vortex-light beams and matter has emerged as a crucial research area with potential far-reaching applications. These beams possess the unique characteristic of carrying a nonzero projection of the orbital angular momentum (OAM) onto the propagation direction. This additional degree of freedom has opened up an exciting opportunity to gain a deeper understanding of the role played by OAM in light-matter interactions. The possible applications of vortex-light beams are numerous, including but not limited to optical manipulation^1,2^, quantum information^3,4^, imaging and microscopy^5,6^, and super-resolution optical sensing^7^. To fully harness their potential, a deeper understanding of twisted light interacting with quantum descriptions of matter is mandatory. Numerous considerable theoretical and experimental investigations have been performed in this direction, encompassing processes such as excitation^8–15^, ionization^16–20^, high harmonic generation^21–23^, and various others. Consequently, there is a growing demand for developing compact and reliable techniques to generate twisted beams that can fulfill the requirements of these emerging photonic technologies. Although the generation of these beams using spiral phase plates^24^, forked holograms^25^, and q-plates^26^ has become commonplace, their bulky configurations make miniaturization and integration difficult. However, recent advances in nano-scale metallic structures, such as Archimedean spirals, offer promising ways to generate on-chip twisted light, providing a new avenue for research in this field^27^.

Up to now, plasmonic Archimedean spirals have been intensively studied for the generation of so-called vortex surface plasmon polaritons (VSPP), hybrid light-matter states on the surface of a metallic structure. The first experimental realization of VSPP was performed by Gorodetski and colleagues^28^. Later on, several works demonstrated setups for synthesis^29^, real-time observation^30^ and beaming^31^ of twisted light at the nanoscale. Finite-difference time-domain (FDTD) simulations have shed light on the mechanism for such twisted polaritonic modes generation^32–34^. However, it has been shown that not only the surface mode of scattered light can possess vortex properties but also its component in the vacuum region^27,35^. Therefore Archimedean spirals can be perfect candidates for the generation of twisted light.

Here, we investigate the generation of an optical vortex beam via the scattering of an ultrashort laser pulse on a gold nanoplasmonic spiral. To do so, we perform real-time, real-space simulations of this process and examine the dynamics of the space-dependent OAM of the scattered pulse. To get a deeper microscopical insight into the mechanism of the formation and control of the vortex beam, we propose an analytical formula for the current that generates a similar vortex. Finally, we study the interaction between a diatomic molecule, modeled as a system of classically described point-like particles, and scattered radiation. We model, for the first time, the complete process from the formation of the vortex beam up until the angular momentum transfer to the point-like particles. Our findings show that the trajectory of the particles strongly depends on their initial position, acting as a probe of the local OAM of the beam.

## Methods

We have performed the real-time propagation of Maxwell’s equations using the real-space, real-time code Octopus^36^. This package, originally used for quantum dynamics and the study of light-driven phenomena, has been adapted to work as a multi-system, multi-physics framework. The implemented approach allows us to couple electromagnetic fields not only with simplified semiclassical models of matter, but also self-consistently with first-principles matter descriptions, within the time-dependent density functional theory (TDDFT) formalism^37^. We emphasize that in this work, we only make use of classical susceptibility models to describe the plasmonic material (*vide infra*). While the interaction of quantum light with nanoplasmonic media^38^ and single apertures^39^ has been reported in the literature, we note that our treatment only considers classical electromagnetic fields. The capability of the Octopus code to also treat quantum mechanical models of matter self-consistently coupled to electromagnetic fields will be subject of future studies.

The computational method is based on the Riemann-Silberstein (RS) representation of electromagnetic fields to propagate Maxwell’s equations in real time. The RS vector is defined as

1 $$\begin{aligned} \vec {F}^\pm (\vec {r}, t) =\sqrt{\frac{\varepsilon _0}{2}} \vec {E}(\vec {r}, t) \pm i \sqrt{\frac{1}{2\mu _0}} \vec {B}(\vec {r}, t). \end{aligned}$$

Here, $\varepsilon _0$ and $\mu _0$ are the vacuum permittivity and permeability, respectively, while $\vec {E}(\vec {r}, t)$ and $\vec {B}(\vec {r}, t)$ are the electric and magnetic field. With this definition, the Ampère and Faraday laws can be combined into one equation of motion of the Riemann-Silberstein vector

2 $$\begin{aligned} i \partial _t \vec {F}^\pm (\vec {r}, t) = \pm c_0 \nabla \times (\pm \vec {F}^\pm )(\vec {r}, t) - i \sqrt{\frac{1}{2\varepsilon _0}}\vec {J}_{total}(\vec {r}, t), \end{aligned}$$

where $\vec {J}_{total}(\vec {r}, t)$ is the total current density. For linear media, this can be written as $\vec {J}_{total}(\vec {r}, t) = \vec {J}_{M}(\vec {r}, t) + \vec {J}_{P}(\vec {r}, t) + \vec {J}_{free}(\vec {r}, t)=\nabla \times \vec {M}(\vec {r}, t) + \partial _t \vec {P}(\vec {r}, t) + \vec {J}_{free}(\vec {r}, t)$, $\vec{M}(\vec {r}, t)$ and $\vec{P}(\vec {r}, t)$ being the magnetization and polarization, respectively. We are concerned with non-magnetic materials in the linear regime, therefore we consider the currents arising from the polarization (in simplified form) $\vec {P}(\vec {r}, \omega ) = \varepsilon _0 \chi (\vec {r}, \omega ) \vec {E}(\vec {r}, \omega )$, where $\chi$ is the scalar (isotropic) electric susceptibility. In particular, we consider gold nanostructures that we model using the standard and widely used Drude susceptibility with pole frequency $\omega _p$ and inverse relaxation time $\gamma _p$

3 $$\begin{aligned} \chi (\omega ) = - \frac{\omega _p^2}{\omega ^2 - i\omega \gamma _p}. \end{aligned}$$

This susceptibility is applied only inside the Drude material, while the vacuum permittivity applies in the external points. This creates a discontinuity in the space-dependent permittivity $\varepsilon (\vec {r},\omega )$ which induces surface charges and currents. We propagate Eq. (2) using the methods described in previous works^37^. We also include point-like particles as test charges in some simulations. Their evolution is simulated with an exponential midpoint scheme^40^. In addition, we solve the auxiliary differential equation for the induced current density in the time domain^41^ using a Runge-Kutta scheme

4 $$\begin{aligned} \partial _t \vec {J}_{P}(\vec {r}, t) + \gamma _p \vec {J}_{P}(\vec {r}, t) = \varepsilon _0 \omega _p^2\vec {E}(\vec {r}, t). \end{aligned}$$

**Figure 1:**
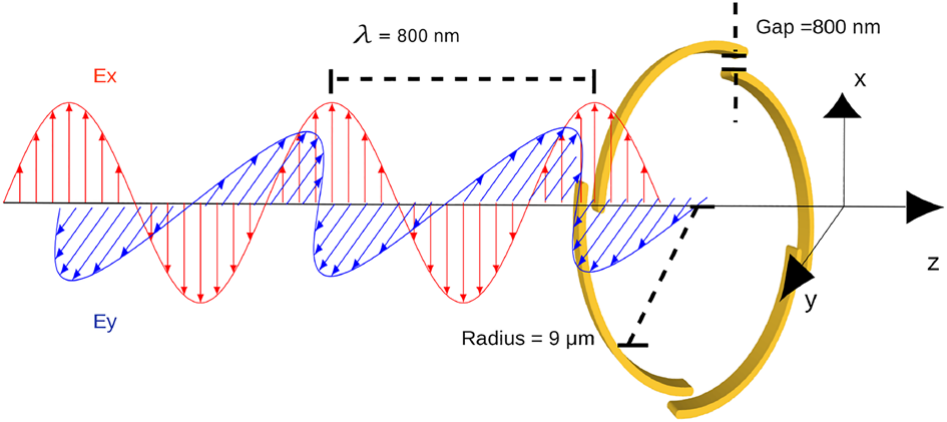
We send an 8-cycle ultrashort right-circularly-polarized pulse with $\lambda = 800$ nm onto the gold, segmented Archimedean spiral with four branches. The gap between the branches is set to 800 nm to match the wavelength, the radius is 9 ${\mathrm {\mu m}}$ and the thickness of the spiral in the propagation direction is 1.6 ${\mathrm {\mu m}}$.

Our simulation setup is shown in Fig. 1. We model the spiral as a linear medium with a Drude susceptibility, using previously reported parameters^42^. The spiral structure is designed and rendered using the computer-aided design program OpenSCAD, and is then read in Octopus using the CGAL library^43^. The employed Archimedean spiral’s shape in polar coordinates is given by

5 $$\begin{aligned} r(\varphi )=r_{0}+\frac{d \bmod (m \varphi , 2 \pi )}{2 \pi }. \end{aligned}$$

Here $r_{0} = \sqrt{x_0^2 + y_0^2}$ marks the starting radius of the segmented Archimedean spiral branches, which expand from there around the center until the radial distance reaches $r_{0} + d$; $\varphi = \arctan (\frac{y}{x})$ is the azimuthal angle and mod is the modulus function giving the remainder of the division of $m \varphi$ by $2\pi$. The number of segments of the spiral is determined by the parameter *m* (for this study, we considered $m=4$).

**Figure 2:**
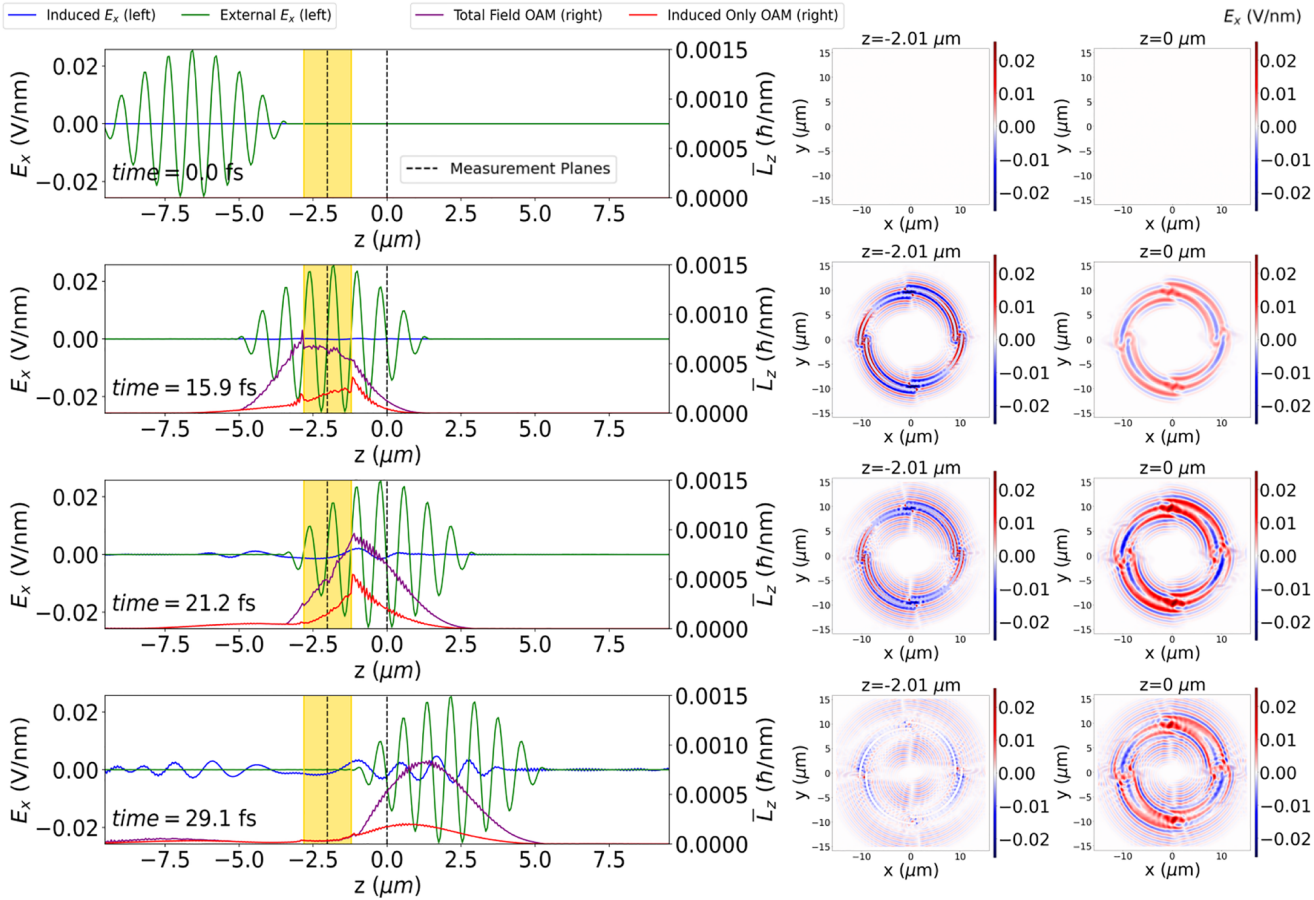
A side view of the simulation box (along the propagation axis) is shown in the left panels, while the corresponding cross-section profiles can be seen on the right. The four rows correspond to four snapshots in time at 0.0 fs, 15.9 fs, 21.2 fs, and 29.1 fs, respectively. The external and induced electric field $E_x$ components are shown in green and blue lines, respectively. The *xy*-integrated OAM density is shown for two cases: the OAM density resulting from total (induced + external) fields is marked in purple, while the OAM density resulting only from the induced field is shown in red. The vertical dashed black lines in the left panels mark the planes for which the cross-section plots are shown in the right panels, namely at the middle of the spiral ($z=-2 {\mathrm {\mu }}$m) and a further plane ($z=0$). After the external pulse arrives at the spiral, we observe that emitted radiation from the structure is traveling towards its center, which is clear from the cross-section of the emitted field at 15.9 fs, 21.2 fs, and 29.1 fs. The OAM density shows that the vortex-like nature of the induced radiation is not only present in the near field but is part of the transverse component that radiates far from the structure.

A right-circularly-polarized ultra-short pulse of 8 cycles of the form

6 $$\begin{aligned} \vec {E}(\vec {r},t)= {\textrm{Re}} \{ g( \vec {r}) e^{i( {\vec { k}}\cdot {\vec {r}}-\omega t)}\vec {\varepsilon }_{\vec {k} \sigma } \}, \end{aligned}$$

is sent toward the nanoplasmonic structure, where $$\vec {\varepsilon }_{\vec {k} \sigma } = \frac{1}{\sqrt{2}}\left( \begin{array}{c}1 \\ \sigma i \\ 0\end{array}\right)$$ is the polarization vector for circularly polarized light with a helicity $\sigma$ (in our case, $\sigma = 1$), and $g( \vec {r})$ is the Gaussian-shaped envelope function, described as

7 $$\begin{aligned} g(\vec {r})= E_0 \exp\left(- \frac{(\vec {k} \cdot ( \vec {r} - \vec {r}_\mathrm{off} ))^2 }{2|\vec {k}|^2 w^2} \right), \end{aligned}$$

where *w* denotes the pulse width in space, which is set as $4\lambda$ in the present study, with $\lambda = 800$ nm. Moreover, $\vec {r}_\mathrm{off}$ marks the offset from the origin of the pulse, which is set such that at $t=0$ the pulse starts already inside the simulation box, as depicted in Fig. 2 (*vide infra*). The simulation box size is 15.9 $\times$ 15.9 $\times$ 10.6 ${\mathrm {\mu m}}$ in size, and the discretization spacing is 52.9 nm. The time step used for the simulation is $2.65 \times 10^{-3}$ fs. A perfectly matched layer (PML) is used to model absorbing boundary conditions. The maximum pulse amplitude $E_0$ is 0.02 V/nm, ensuring the validity of the linear regime.

## Results

### Evolution of orbital angular momentum in time

The time-resolved dynamics of the interaction of the circularly-polarized pulse with the spiral is analyzed by decomposing the total electromagnetic fields in their external and induced components: $\vec {E}(\vec {r}, t) = \vec {E}^{{\textrm{ext}}}(\vec {r}, t) + \vec {E}^{{\textrm{ind}}}(\vec {r}, t)$. The external pulse propagates in a vacuum and is not scattered by the medium, while the induced component contains all the relevant effects from the spiral. Further insight is provided by the space-dependent OAM density, which is calculated from the total $\vec {E}(\vec {r}, t)$ and $\vec {B}(\vec {r}, t)$ fields as follows^44^

8 $$\begin{aligned} \vec {L}(\vec {r}, t) = \frac{1}{c^{2}\mu _{0}} \vec {r} \times \left[ \vec {E}(\vec {r}, t) \times \vec {B}(\vec {r}, t)\right] = \varepsilon _{0} \vec {r} \times \left[ \vec {E}(\vec {r}, t)\times \vec {B}(\vec {r}, t)\right] . \end{aligned}$$

For visualization purposes, the *xy*-plane averaged OAM density $\bar{L}_{z}(z,t)$ is obtained by

9 $$\begin{aligned} {\bar{L}}_z(z, t) = \frac{1}{S_{xy}} \int \int |L_z(x,y,z,t)| dx dy, \end{aligned}$$

where $S_{xy}$ is the cross-sectional area of the simulation box. We now analyze the cross profile of the electric field and average OAM content ${\bar{L}}_z(z,t)$, as depicted in Fig. 2. There, the side view of the spiral is shown as the gold shaded area, and the profile of the external field $E_x^{{\textrm{ext}}}(\vec {r}, t)$ component through the *z*-axis is shown in green while the induced electric field $E_x^{{\textrm{ind}}}(\vec {r}, t)$ along the same axis is in blue. As the induced field here is shown only along the propagation axis, the dynamics towards a vortex-like structure is better explained by the averaged OAM density ${\bar{L}}_z(z, t)$ along the *z*-axis, which is shown in purple in Fig. 2. Following its definition in Eq. 8, we calculate the OAM density considering the total electric and magnetic fields (external and induced). For comparison, we also plot the OAM density stemming from only the induced fields, in red. The comparison between the two OAM densities indicates that the interference of the incoming circularly polarized field with the emitted field contributes significantly to the OAM density, the amplitude of the one resulting only from induced fields being much smaller, with a profile that follows the regions of maximal induced field intensity (e.g. near-field peaks at the surface of the nanostructure, and decreasing amplitude as it travels further from the spiral). The OAM density from the total fields does not remarkably change in amplitude after the external pulse interacts with the nanospiral, as its average profile is mostly governed by the amplitude of the source pulse, but showing a delay with respect to the external pulse envelope due to the emitted free-space fields. Another quantity that demonstrates the contribution of the external fields in the amplitude is the averaged energy density along the propagation axis (see Supporting Information).

The *xy* dependence of the induced electric field $E_x^{{\textrm{ind}}}(\vec {r}, t)$ component is shown on the right panels of Fig. 2, each row corresponding to the time snapshot of the left panel, and the planes of measurement marked with dashed lines also on the left panels (one plane located in the middle of the spiral, the other one at the $z = 0$ plane of the simulation box). The emitted field emanates from the branches and travels toward the center of the spiral, interfering at the central axis of the structure. However, in addition to the field localized at the spiral plane, the emission of the spiral is significant a few micrometers away from its position, and such an induced field carries a non-negligible OAM density, as seen in the snapshots at 21.2 fs and 29.1 fs. This traveling component could be better observed in the cross-section $E_x^{{\textrm{ind}}}$ plots on the right panel at $z=0$ (2 ${\mathrm {\mu }}$m away from the spiral), where it is clear that the profile resembles the one at the spiral plane but with a certain delay. The role of the circular polarization in the incident beam, as well as the mechanism for OAM generation by scattering from the branches, can be better understood by a more simplified model, as explained in the next section.

### Emulating the emission with structured current emitters

To explore the mechanistic origin of the features of vortex-like radiation, we model in this section the process as a Maxwell simulation in vacuum with external current sources mimicking the full emission from the medium. This helps us to develop a microscopic understanding of the phenomenon which could be relevant for future works where matter and electromagnetic fields are both treated ab initio. This modeling also allows us to run more efficient simulations without the need to explicitly include the linear medium, which demands a much finer mesh to properly describe the field dynamics on the surface of the object and alleviates the spurious fields produced at specific locations such as the caps around the edges of the spiral. The fact that the OAM density amplitude depends also on the external fields does not invalidate this approach based on mimicking only the induced fields, since the OAM densities calculated from total and induced fields are very similar in the region closer to the center of the spiral (*z*-axis), and it is governed by the emitted pulse, as evidenced from the cross-sectional patterns (see Supporting Information).

We prescribe the spatial and temporal profile of the current density emitters, shaped as a segmented Archimedean spiral in the simulation box to emulate the field produced by the plasmonic spiral, according to Eq. (5). The *x* and *y* components of the emitter’s current distribution are given by

10 $$\begin{aligned} J_{x}(x,y,z,t) = j_{0} \exp \left( \frac{-\left( \sqrt{x^{2}+y^{2}} - r_{0} - d \varphi \right) ^{2}}{{2\sigma'^{2}}} \right) \exp \left( \frac{-(t- t_{0})^{2}}{2\tau ^{2}} \right) \cos (\omega t), \end{aligned}$$

11 $$ J_{y}(x,y,z,t) = j_{0} \exp \left( \frac{-\left( \sqrt{x^{2}+y^{2}} - r_{0} - d \varphi \right) ^{2}}{{2\sigma ^{\prime}{2}}} \right) \exp \left( \frac{-(t- t_{0})^{2}}{2\tau ^{2}} \right) \cos (\omega t + \pi /2). $$

Here *d* represents the gap between the branches of the segmented spiral. $j_{0}$ is the current density amplitude and is set to 7.57 $\times 10^{-7}$ A nm$^{-2}$ in our simulations. The spiral shape is described by four segments (from 0 to $\frac{\pi }{2}$, $\frac{\pi }{2}$ to $\pi$, $\pi$ to $\frac{3}{2}\pi$ and $\frac{3}{2}\pi$ to $2\pi$). In the spatial domain, a Gaussian envelope with width $\sigma'$ of 0.08 ${\mathrm {\mu }}$m is introduced to smooth out the signal. The temporal signal is a cosinoidal carrier with frequency $\omega$ of 1.55 eV and a Gaussian envelope in time, as seen in Eqs. (10) and (11), with parameters $t_{0} = 13.3$ fs, which denotes the time shift, and $\tau = 4$ fs is the width of the pulse in time.

**Figure 3:**
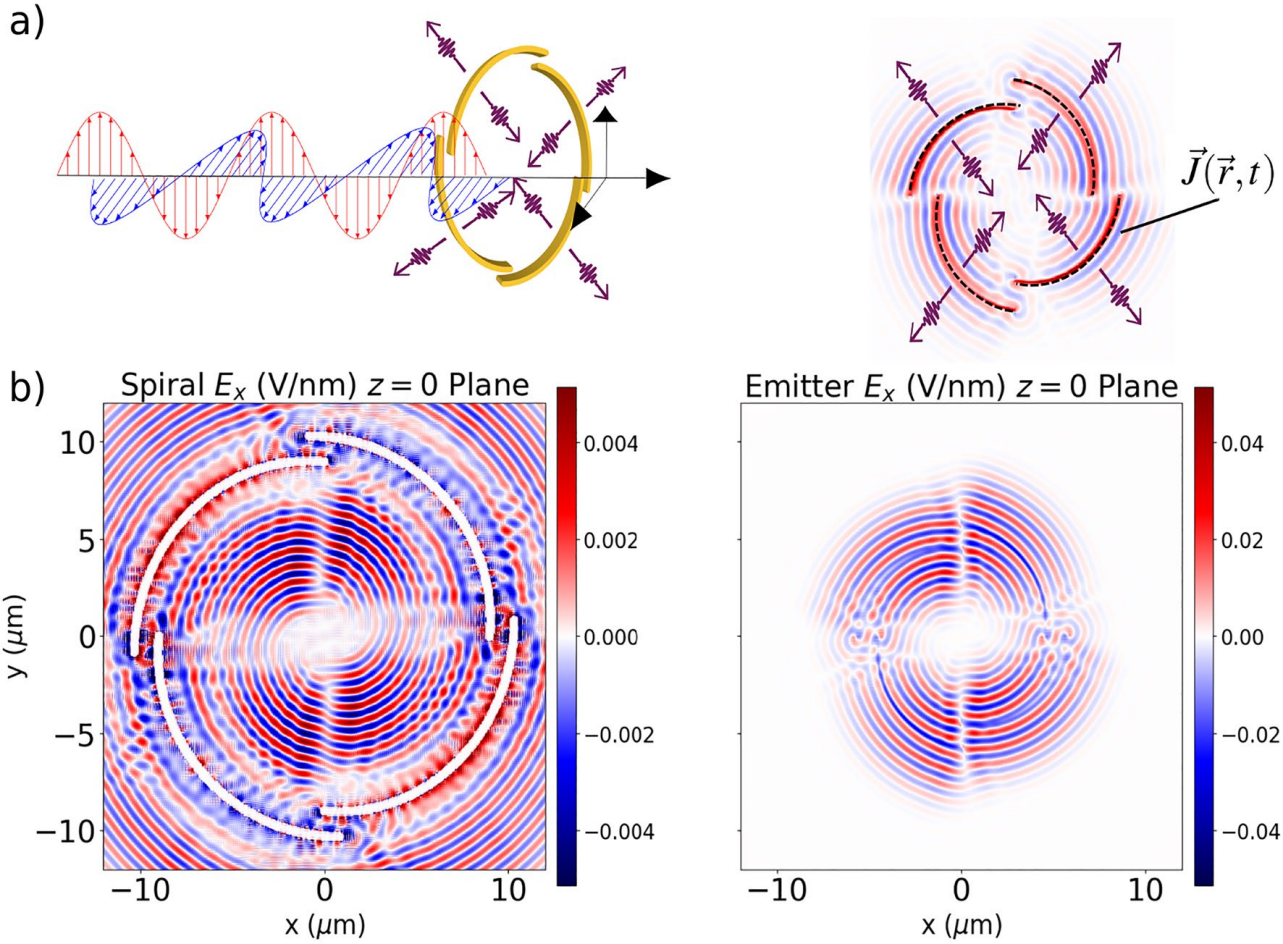
The figure compares the induced electric field from the spiral described as a Drude medium (left side) with the emission that emerges from the current density in Eq. (10) (right side). The panel (**a**) describes the schematics of the setups for the two different approaches: the nanoplasmonic spiral on the left hand side, and the current emitters, which are prescribed directly in simulation box (dashed black lines) on the right hand side. In panel (**b**), we present the cross sectional profile of both approaches. For computational reasons, we set the radius of the spiral emitter 4.5 ${\mathrm {\mu }}$m as half of the radius of the actual spiral (9 ${\mathrm {\mu }}$m).

In Fig. 3 the two mechanisms for vortex generation, namely the plasmonic spiral and the current emitters, are compared. For the emitters (right panel), the spiral radius $r_0$ was set to 4.5 ${\mathrm {\mu }}$m, which is half of the radius of the actual spiral (left panel), for computational efficiency. Even when the sources are located at a different distance from the origin, both patterns show great resemblance with the same number of nodal lines and interference profile, although a quantitative comparison is precluded by the structural differences between the plasmonic spiral (which has a finite thickness) and the current emitters (much smaller thickness, defined by the parameter $\sigma'$). The validity of the model spiral was also checked throughout time, both for electric field amplitude and OAM density (see Supporting Information). However, even in the case of the perfect spiral-shaped emitters, which do not have the numerical inaccuracies present in the description of radiation at the surface of metals, the cross-sectional plot does not immediately resemble a Bessel-beam like structure, as reported in previous works in the frequency domain^35^. The differences can arise from the broad frequency spectrum of such an ultrashort pulse, which creates spurious signals that preclude the formation of a perfect nodal structure with the appropriate number of phase discontinuities. A systematic study increasing the length of the pulse to approach the continuous-wave regime would be necessary to confirm this hypothesis, although it is much more computationally demanding.

### Probing the OAM with a test charge

We now use two point-like charged particles to probe the transfer of OAM from light to matter at different points in space with full-time resolution. From the computed trajectory, we can evaluate the angular momentum change that the particle experiences through the interaction with the electromagnetic field.

The two point-like particles that we employ in the simulations are bound by a Lennard-Jones potential with parameters $\varepsilon _{{\textrm{LJ}}} = 2.72$ eV, $\sigma _{{\textrm{LJ}}} = 0.053$ nm, and have opposite charges of ± $1.602 \times 10^{-19}$ C to ensure overall charge neutrality. One of the particles has a much heavier mass ($9.1\times 10^{-21} g$ , $10^7$ $m_e$) while the other has a much lighter mass of 1 u ($1.67\times 10^{-24} g$ , 1836 $m_e$, 1 atomic mass unit). As a result, the second particle can be regarded as the probe as it is allowed to orbit the heavy particle, which serves as an anchor. The 2-particle system is studied in two simulations: in one case, the system is subjected to the field emitted by a spiral emitter, while it interacts with a plane wave in the other. The plane wave simulation serves as a reference and uses a right circularly polarized field. The particles interact with light via the Lorentz force

12 $$\begin{aligned} \vec {F}(\vec {r}, t) = q(\vec {E}(\vec {r}, t) + \vec {v} \times \vec {B}(\vec {r}, t)), \end{aligned}$$

where *q* is the charge of the particle and $\vec {v}$ its velocity. By placing the particle system at different initial locations in the *xy* plane of the spiral emitter simulation, we study the local angular momentum transfer and relate it to the local properties of the field. We calculate the induced angular momentum change of the particle $\vec {L}_p$ as follows

13 $$\begin{aligned} \vec {L}_p = m\left[ (\vec {r}-\vec {r}_\mathrm{init})\times \vec {v}\right] , \end{aligned}$$

where *m* is the mass, $\vec {r}_\mathrm{init}$ and $\vec {r}$ are the initial and time-dependent position, respectively.

**Figure 4:**
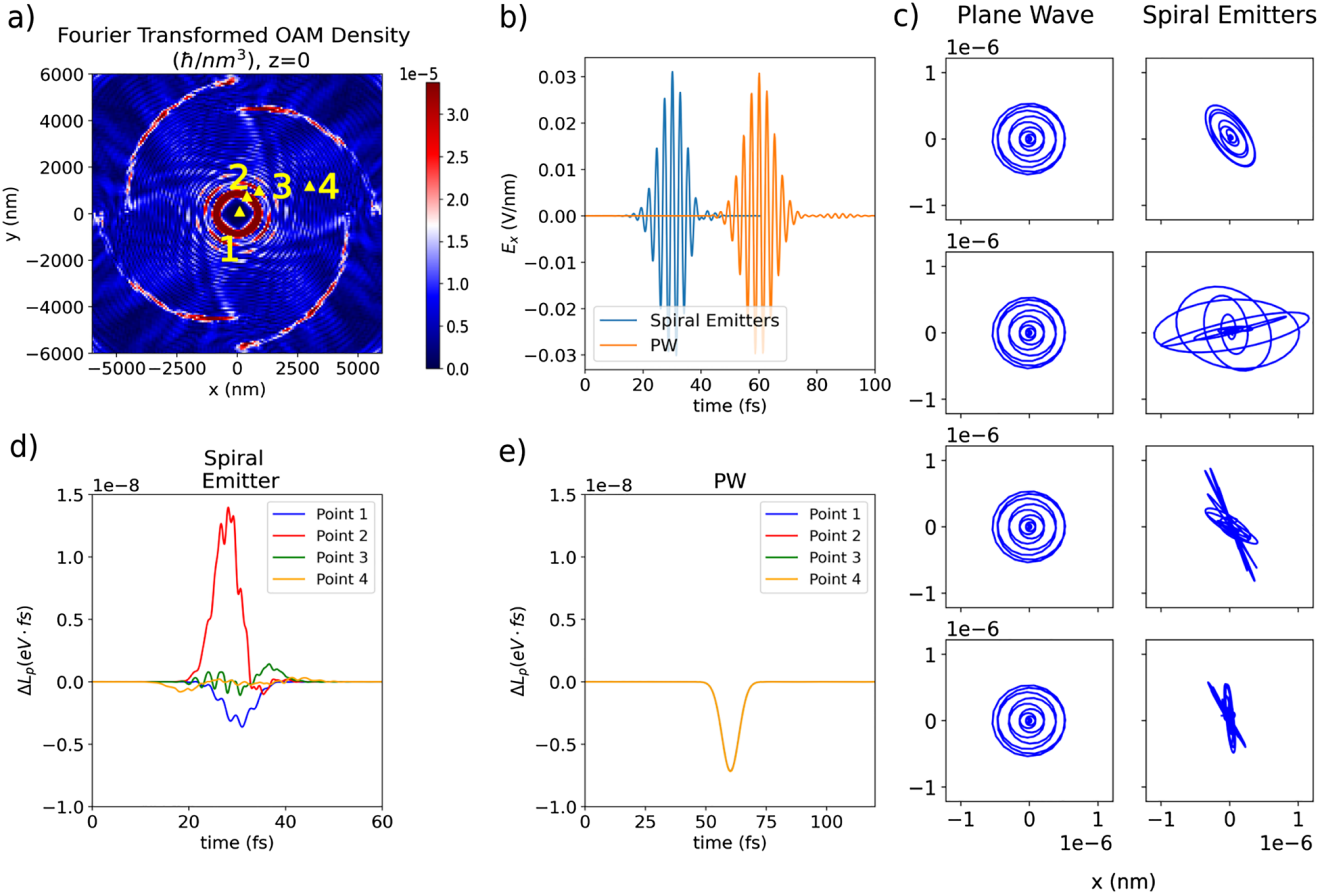
We probe the generated structured light field by placing the point particles at different locations. In panel (**a**) we present the Fourier component of the orbital angular momentum density (8) at the $z=0$ plane, at the frequency of 1.55 eV which corresponds to 800 nm wavelength we employ in scaled-down simulation. The yellow triangles in this plot point to locations 1–4 of the probe particles which are modeled as a diatomic molecule. These points mark the *x* and *y* coordinates of both particles, the lighter one is located at the $z=0$ plane and the heavier (anchor) particle resides in the $z=-d_{eq}$ plane, where $d_{eq}$ denotes the Lennard-Jones equilibrium distance of 0.065 nm between the particles. In (**b**) the temporal behavior of the electric field $E_x$ emitted by a spiral-shaped source and right circularly-polarized plane wave at point (0,0,0) is presented, showing the same amplitude and only a delayed arrival of the plane wave pulse to the location of the particle. Here, both the pulse emitted from the spiral emitter and the plane wave have a full width at half maximum (FWHM) of 0.3 eV. In (**c**) the *xy*-trajectories of the lighter particle are shown in the presence of a plane wave pulse (left column) and radiation generated by spiral emitters (right column). In both columns, the labels 1-4 correspond to particle positions 1-4 in panel (**a**). In (**d**,**e**) *z* component of the angular momentum change $L_{p}$ as described in Eq. (13), for particles at locations 1–4 from panel (**a**) in the presence of the vortex-like beam (**d**) and the plane wave pulse (**e**) are shown. Here, the reported quantity is denoted as $\Delta L_{p}$ which is the difference in angular momentum since the particle starts at rest with zero angular momentum.

In Fig. 4 we analyze the interaction between the point particles and the different light sources. To depict the OAM density of the beam in the *xy* plane in an efficient way, we compute the Fourier transform of the time-dependent orbital angular momentum density of the vortex-like emitted field in the simulation box according to

14 $$\begin{aligned} \tilde{\vec {L}}(\vec {r},\omega ) = \int ^{+\infty }_{-\infty } \vec {L}(\vec {r},t) e^{i\omega t}dt. \end{aligned}$$

From the OAM density map shown in Fig. 4 we can identify OAM-density ”hot spots” in the concentric-ring structure produced by the spiral emission. Then, we place the particle system at the positions marked by 1-4 in Fig. 4 a. The particle motion is computed not only under the influence of emitter-spiral shown in Fig. 3, but also induced by a circularly-polarized plane wave, as shown in Fig. 4b. We engineer the plane wave pulse such that the scaled spiral emitter and plane wave pulse have similar amplitudes and envelopes for a fitting comparison. The trajectories of the point particle placed at different locations are shown in Fig. 4c. Here, the initial positions are subtracted to observe the displacement of the particle. The orbiting particle receives the angular momentum change induced both by the spiral emitters and the plane wave pulses in a limited amount of time, producing a circular trajectory and then returning to its initial position. While the plane-wave-induced trajectories remain circular and uniform, the spiral-emitter-induced trajectories are deformed, which is in agreement with previous findings^45^. Movies for the trajectories of the particle system located in point 2 can be found in the Supporting Information.

The change of angular momentum of the point particle $\Delta L_{p}$ is shown for points 1-4 in Fig. 4d,e. On the one hand, as expected, the plane wave pulse conveys the same change in the angular momentum of the probe particle regardless of the location of the particles, while the change of angular momentum is space-dependent for the pulse carrying OAM. On the other hand, the field induced by the spiral-shaped emitter produces different temporal behavior in the angular momentum of the particle depending on the location. By assessing the effect on the different locations in terms of the OAM density of the pulse depicted in Fig. 4a, we note the OAM density is maximum at point 2. However, at point 1 a surprising result is obtained, showing a larger change in angular momentum than the one predicted by the OAM density map which is at a minimum at the vortex center, as seen in Fig. 4a. The angular momentum change of the particle continues to decrease in point 3 and point 4. In this sense, the change of angular momentum produced by the spiral emitter follows the expected trend for points 2, 3, and 4, but at the vortex center this classical OAM density fails to predict the observed effect.

## Discussion

We have studied the generation of orbital angular momentum with nanoplasmonic Archimedean spirals with classical electrodynamics simulations. We employed the classical description of orbital angular momentum^44^, and probed its emergence in the simulation box. We report that the OAM density profile has a clear spatial and temporal structure, similar to the expected profile obtained in previous studies^35^, but with differences due to the time envelope of the incident beam. We confirm the generation of a vortex-like structure able to transfer angular momentum to a classical particle system with the full spatial and temporal resolution, using a scaled-down model of the source.

When located at different points situated on the cross-sectional profile of the beam, the trajectories and the change of angular momentum for these particle systems are altered. The classical local OAM density agrees with the transferred angular momentum for all points except the one at the vortex, which is where the non-trivial effects of light with OAM are expected. This calls for further work to clarify the validity of a classical description of angular momentum for light in this regime. In this sense, we intend to use the same framework to model the interaction of vortex beams with the matter described from first principles, by coupling self-consistently TDDFT with Maxwell’s equations in real-time, and accounting for the full space dependence of the electromagnetic fields via the full minimal coupling Hamiltonian^37^. The coupling of analytical beams with OAM (e.g. Bessel beams) as well as fields produced by plasmonic optical vortex generators with electronic systems opens the door for novel spectroscopy techniques going beyond the dipole approximation and addressing momentum-resolved phenomena with optical pulses in addition to the already explored electron and neutron spectroscopy techniques. Finally, the use of structured fields with OAM inside cavities, by solving Maxwell’s equations in the frequency domain in cavities with active mirrors, could enable breaking additional symmetries beyond the time-reversal symmetry already broken with circularly polarized light in materials^46^, unlocking the potential for light-matter interactions in the strong coupling regime with OAM, with exciting applications in non-equilibrium quantum materials.

### Supplementary Information

Supplementary Information.

## Data Availability

The datasets generated and analysed during the current study are accessible in this repository (DOI:10.5281/zenodo.8208674).
