# Supplementary material for: Time-resolved plasmon-assisted generation of optical-vortex pulses
Source: Sci Rep. 2023 Sep 7;13:14748. doi: 10.1038/s41598-023-41606-3 (PMC10484912; doi:10.1038/s41598-023-41606-3)
Supplement: Supplementary file 1 — Supplementary Information. [file 41598_2023_41606_MOESM1_ESM.pdf]

Supporting Information for "Time-Resolved Plasmon-Assisted Generation of Optical-Vortex Pulses"

Esra Ilke Albar[1], Franco P. Bonafé[1,*], Valeriia P. Kosheleva[1], Sebastian T. Ohlmann[2], Heiko Appel[1], and Angel Rubio[1,3,4,+]

[1]Max Planck Institute for the Structure and Dynamics of Matter, Center for Free Electron Laser Science, Luruper Chaussee 149, 22761 Hamburg, Germany
[2]Max Planck Computing and Data Facility, Gießenbachstr. 2, 85748 Garching, Germany
[3]Center for Computational Quantum Physics (CCQ), The Flatiron Institute, 162 Fifth Avenue, New York, New York 10010, USA
[4]Nano-Bio Spectroscopy Group, Departamento de Física de Materiales, Universidad del País Vasco, 20018 San Sebastian, Spain
[*]franco.bonafe@mpsd.mpg.de
[+]angel.rubio@mpsd.mpg.de

S1 Electric field and OAM density evolution from plasmonic spiral and current emitters

The time evolution of the xy-plane profiles of the induced electric field E_x component and OAM density L_z component are depicted in Figures S1 and S2, respectively. Here, time snapshots every 2.6 fs are ordered from top to bottom for the nanoplasmonic spiral starting from 34.4 fs, highlighting the formation of the vortex-like structure both when an actual spiral as well as when the emulating current density are used. Corresponding emitter snapshots on the right hand side are reported with intervals of 2.3 fs , starting from 23.1 fs .

S2 Energy Density During Propagation

We calculate the energy density ($\vec{W}$):

$$\vec{W}(\vec{r},t) = \frac{\varepsilon_0}{2}\vec{E}(\vec{r},t)^2 + \frac{2}{\mu_0}\vec{B}(\vec{r},t)^2 \tag{1}$$

We integrate it over the cross section seen in Eq. 2 with a similar procedure to OAM and report it in Fig. S3.

$$\bar{W}(z,t) = \frac{1}{S_{xy}}\int\int |W(\vec{r},t)|dxdy, \tag{2}$$

S3 Induced Field OAM compared with Total OAM

We report the comparison of cross sectional OAM densities in Fig. S4. Here, it is visible that emission governs the cross sectional OAM pattern. Therefore, we focus on imitating the emitted field for the mapping of the OAM.

S4 Evolution of point-like particles trajectories

The trajectories for the point-like particles located in "point 2" as shown in in Fig. 4 of the main text are provided as movie files. The corresponding files for the particles can be found here: `https://doi.org/10.5281/zenodo.8208595` (DOI:10.5281/zenodo.8208595).

S5 Computational details

The Octopus code (`https://octopus-code.org`) was used for the simulations. Calculations were run in the cluster Ada of the Max Planck Computing and Data Facility. The code version and commit hash to reproduce the simulations are as follows:

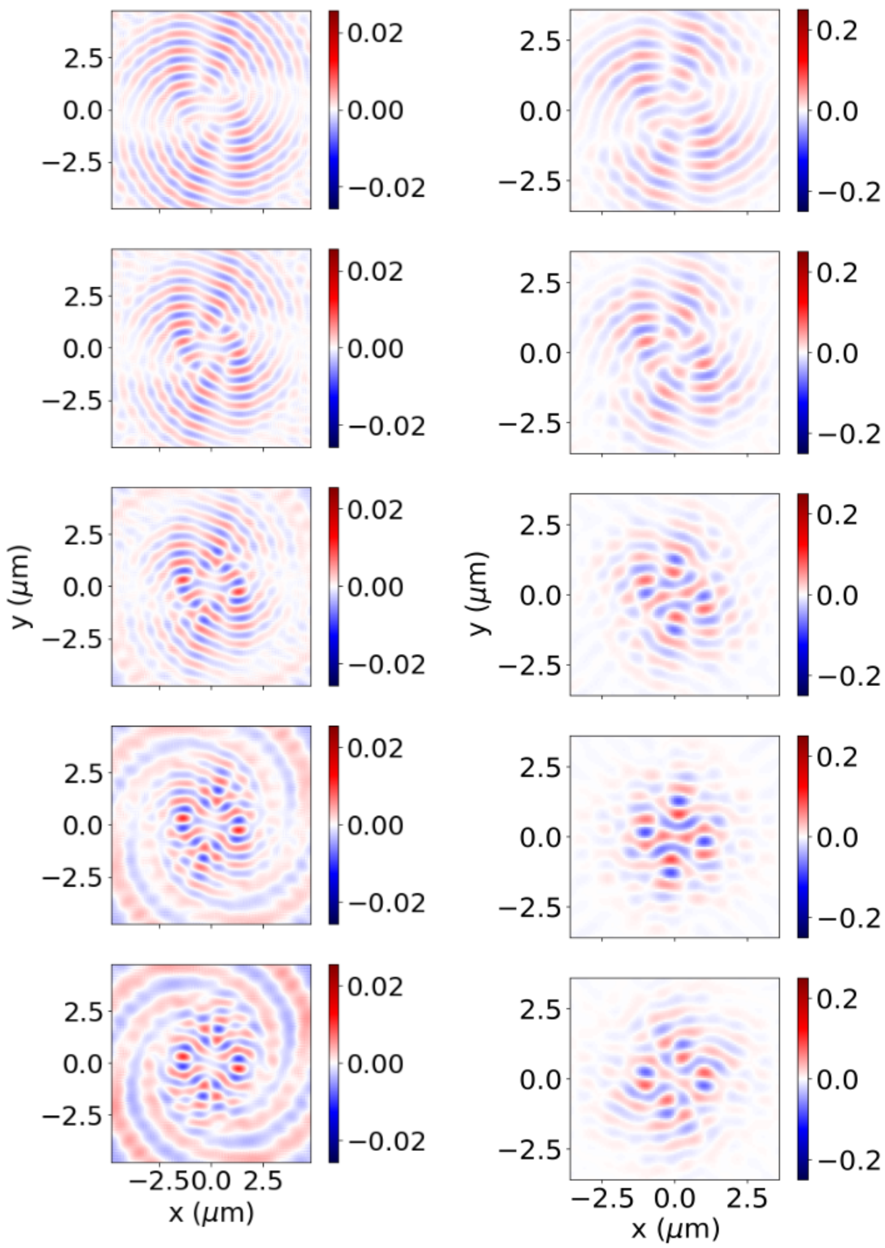

Figure S1. Time dependent evolution of the electric field $E_x(V/nm)$ component provided for the gold spiral (left) and spiral-shaped emitters (right). Time snapshots are 2.6 fs apart starting from 34.4 fs at the uppermost panel for the nanoplasmonic spiral. Corresponding emitter snapshots on the right hand side are reported with intervals of 2.3 fs , starting from 23.1 fs .

```
Version                 : bimaculatus
Commit                  : 00c2a0a716dd1cd5fbae3c9d03a011df131fadd7
```

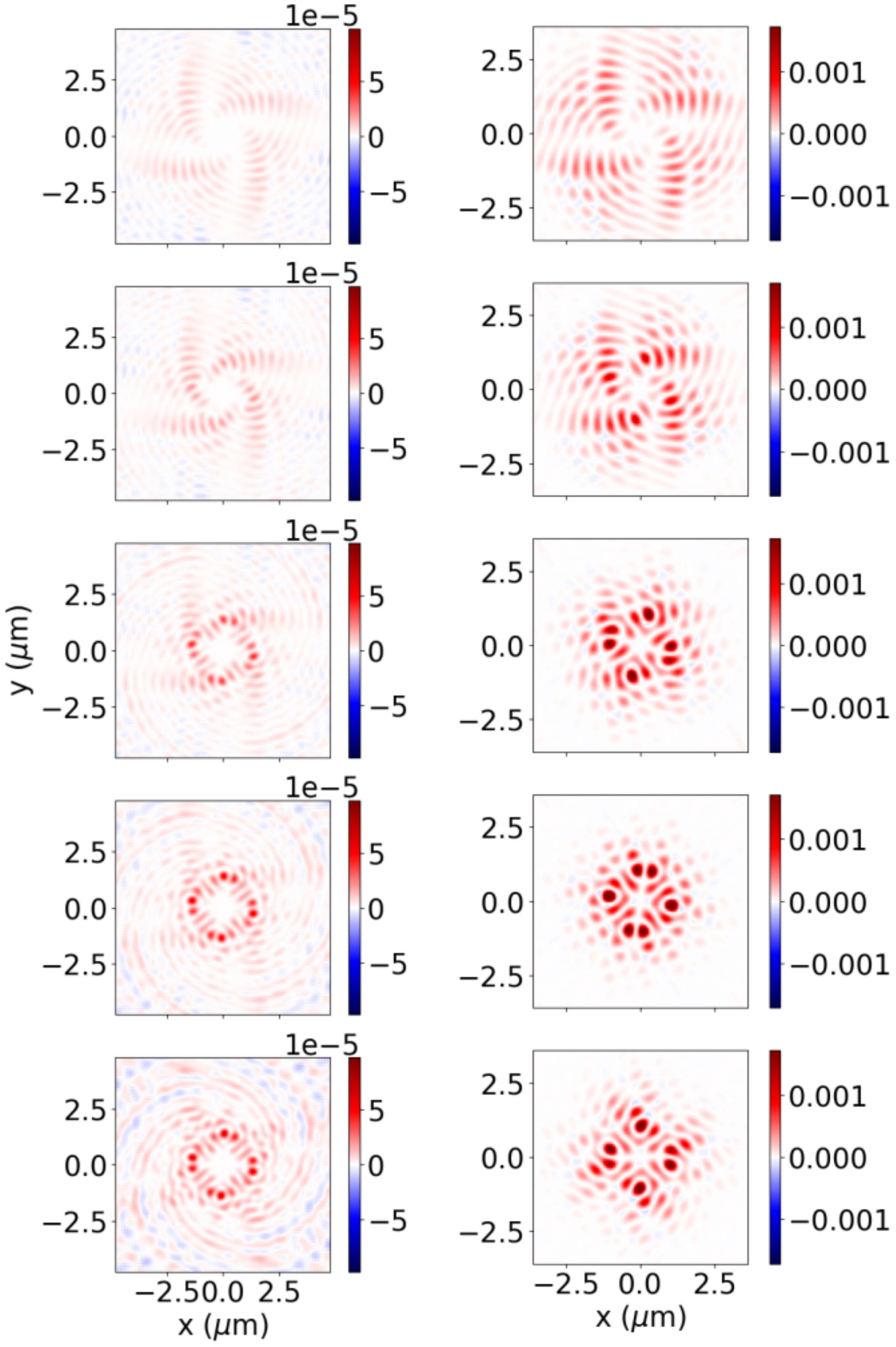

Figure S2. Time dependent evolution of OAM density $L_z(\hbar/nm^3)$ provided for gold spiral (left) and spiral-shaped emitters (right). Here, time snapshots are 2.6 fs apart starting from 34.4 fs at the uppermost panel for the nanoplasmonic spiral. Corresponding emitter snapshots on the right hand side are reported with intervals of 2.3 fs , starting from 23.1 fs .

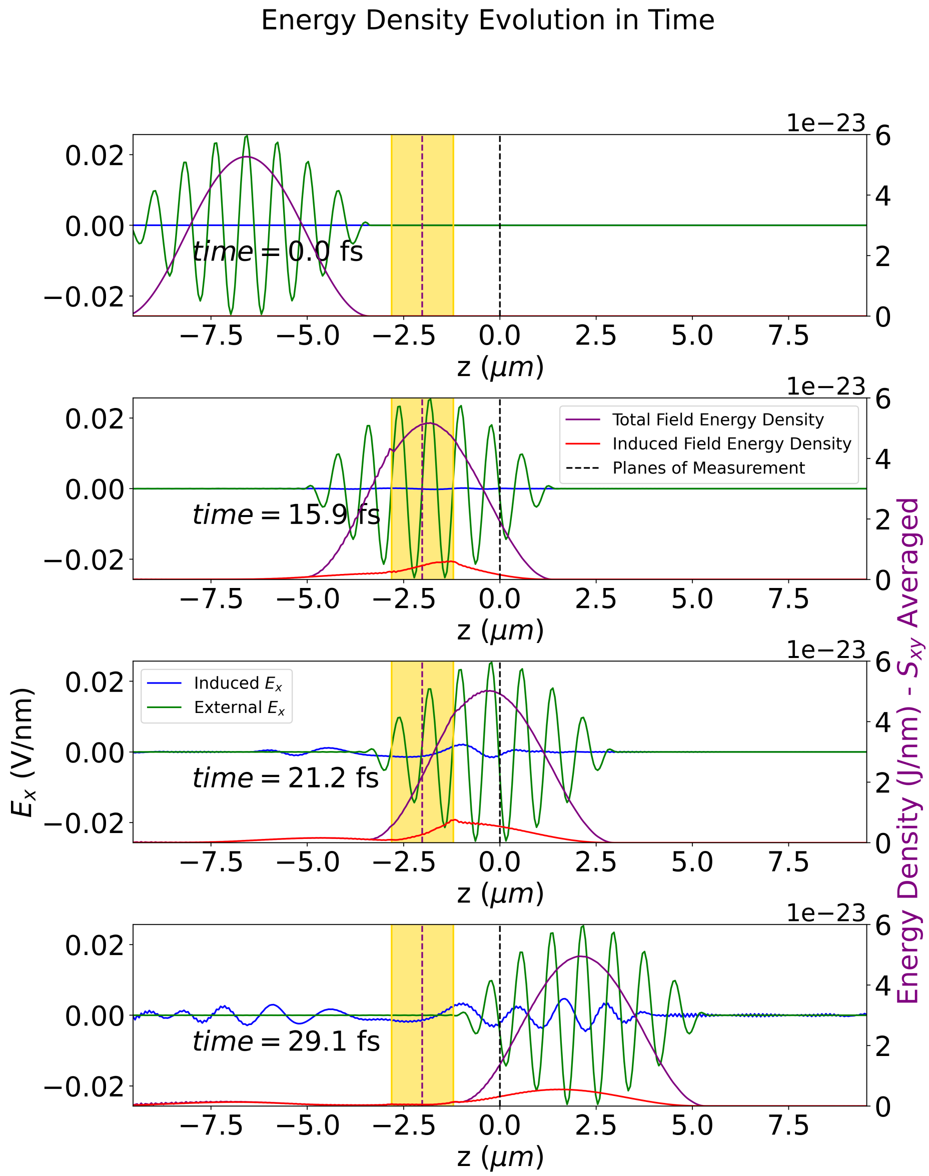

Figure S3. Time dependent evolution of energy density reported along propagation axis. Here, left side indicates the evolution of x component of the electric field while the right hand side of the graph shows that of energy density described in Eq. 1.

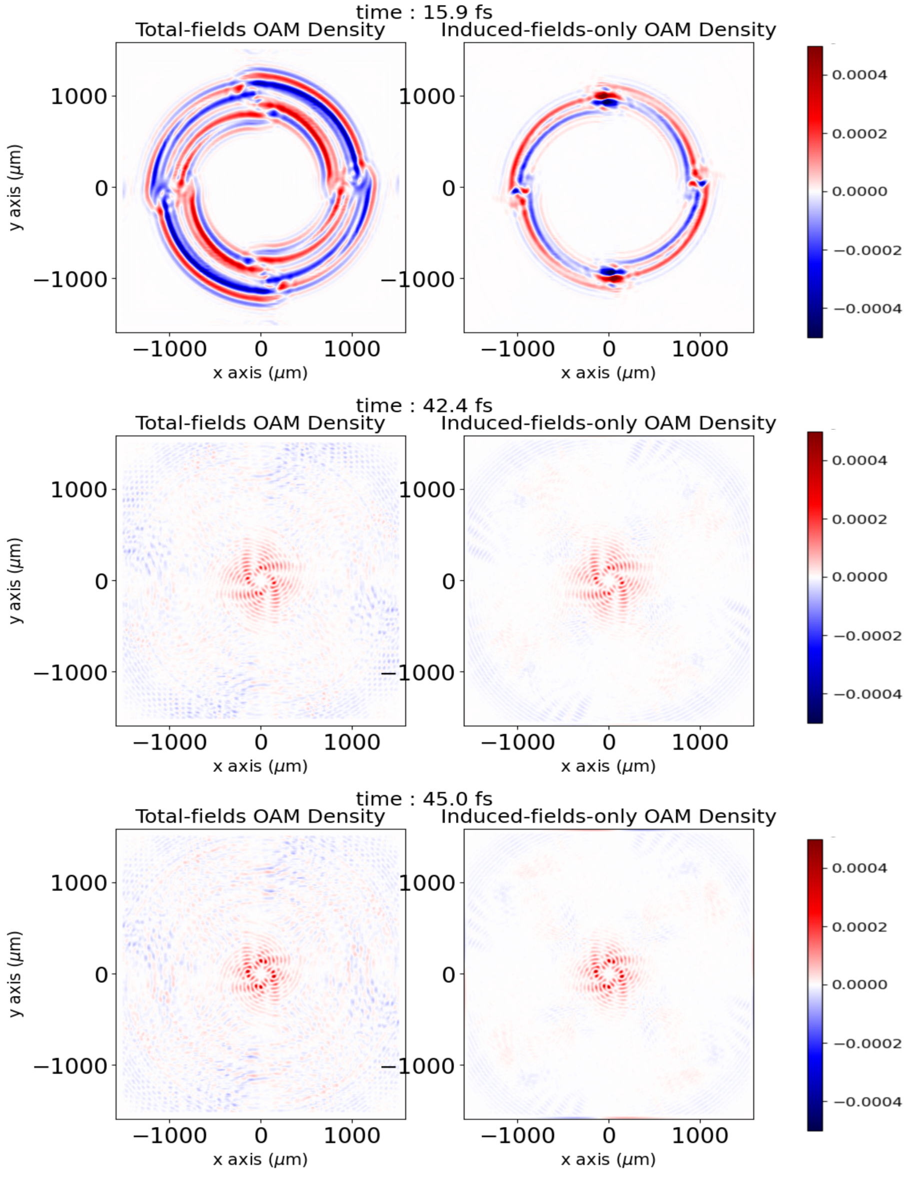

Figure S4. Comparison of cross sectional OAM densities for total fields and isolated induced field.
